# Dietary Pattern among Schoolchildren with Normal Nutritional Status in Navarre, Spain

**DOI:** 10.3390/nu6041475

**Published:** 2014-04-11

**Authors:** Teodoro Durá-Travé, Fidel Gallinas-Victoriano

**Affiliations:** 1 Department of Pediatrics, Faculty of Medicine, University of Navarra; 2 Deparment of Pediatrics, Navarra Hospital Complex, Avenue Irunlarrea, 4, Pamplona 31008, Spain; E-Mail: fivictoriano@hotmail.com

**Keywords:** dietary patterns, food intake, nutritional status, schoolchildren

## Abstract

A nutrition survey was carried out (food intake registration of three consecutive school days) in a randomly selected group of 353 schoolchildren (188 males and 165 females) with normal nutritional status. The average age of the surveyed students was 10.5 years (CI 95%: 10.3–11.7). There were no significant differences between both sexes in mean values for calorie intake (males: 2072.7 ± 261.7 and females: 2060.9 ± 250.6) and intake of macronutrients, minerals and vitamins. Cereals (34%), dairy products (19%) and meats (17%) were responsible for approximately 70% of total calorie intake. Protein accounted for 20.3% of energy intake, carbohydrates for 48.8%, total fat for 30.9%, and saturated fat for 12.6%. Cholesterol intake was excessive and over two-thirds of protein intake was from animal sources. The mean intakes of calcium, iodine and Vitamins A, D and E were below recommended levels. The dietary patterns of the schoolchildren with normal nutritional status differed from the Mediterranean diet. Intakes of meat were too high and dairy products and cereals consumption was relatively limited; while that of vegetables; legumes; fruits and fish were insufficient; leading to excessive protein and fat intake from animal sources and insufficient mineral (calcium and iodine) and Vitamins A; D and E intake.

## 1. Introduction

Dietary habits have always been a social and cultural referent of the different societies. However, scientific evidence in the past years associating diet and health condition has been essential in order to consider dietary habits of a concrete population as a social and sanitary indicator [1]. The nutrition habits traditionally observed in some countries of the Mediterranean area have created the concept of “Mediterranean diet”, whose nutritional interest lies in the variety of foods included in it—in fact, every food group is always provided for in an adequate proportion—and the balanced calorie as well as nutrient intake they guarantee, through a combination of fruits and vegetables with bread, pasta and rice, as well as legumes with dairy products, fish and red meat and olive oil as a cooking fat [2,3,4].

Nevertheless, industrialization and marketing of the food chain in western countries, increasing production of processed food, are leading to a series of changes in feeding habits and preferences in wide sectors of the population. Society has accepted an occidental dietary pattern characterized by an excessive intake of food of animal origin, especially meat and derivatives, and added sugars, at the expense of the intake of cereals, vegetables and fruits. This fact is leading to an increase of saturated fat and cholesterol in diet [5,6,7,8]. This virtual deterioration of dietary patterns in our social and cultural environment has feared a gradual disappearance of Mediterranean diet [9,10] and would justify, to a great extent, the study of the quality of feeding habits in the general population and, especially, in those sectors of population more susceptible of being influenced, such as infant population. On the other side, we assume that schoolchildren with normal nutritional status have feeding habits that guarantee the necessary energy and nutrient requirements—without deficiencies or excess—and that they would contribute to the prevention of adult diseases such as ischemic coronary disease, atherosclerosis, osteoporosis, obesity, diabetes, high blood pressure, *etc.* [11,12,13,14,15,16,17,18,19].

The aim of this work is to achieve a descriptive study of feeding habits in a group of students of primary education with normal nutritional status, as well as to analyze the adequacy of energy and nutrient intake in these schoolchildren to the established nutrition requirements in order to design nutrition intervention strategies.

## 2. Material and Methods

### 2.1. Patients

A nutritional survey has been conducted in a randomly selected group of 353 students (188 males and 165 females) of primary education (3rd–6th grade: 9–12 years old) in the city of Pamplona. All those schoolchildren who suffered from any acute chronic disease which might condition their nutrition status, those who use energy and/or vitamin and mineral supplements and all those students who had lunch out of their own home (home of relatives, school lunch, *etc.*) were excluded. The normality of the nutrition status was the condition sine qua non to be included in this study; this means, BMI should range between +1 and −1 standard deviations (Z-score).

### 2.2. Nutrition Survey

The nutrition survey was carried out in the form of personal interview using a food intake registration of three consecutive school days. Every patient was asked about food intake in every meal during the previous three consecutive days (breakfast, mid-morning snack, lunch, afternoon snack and dinner). A photograph album with portions and measures from the *Institut Scientifique et Technique de la Nutrition et de l´Alimentation* (Paris, 2002) [20] was used to calculate the size of the corresponding portions of the different foods that the participants referred to have eaten.

The foods were divided into the following groups:

1) Milk and dairy products
2) Cereals and cereal products
3) Sweets, bakery and pastry
4) Fruits and natural juices
5) Fats and oils
6) Meat and derivatives
7) Eggs and derivatives
8) Vegetables and tubers
9) Legumes
10) Fishes

Energy and nutrient consumption (proteins, carbohydrates, total fat, SFA, MUFA and PUFA, total fibre and cholesterol), minerals (calcium, iron, iodine, magnesium, zinc, selenium, and phosphorus), and vitamins (thiamine, riboflavin, niacin, Vitamin B6, folate, Vitamin B12, Vitamin C, Vitamin A, Vitamin D and Vitamin E) were calculated using the CESNID 1.0^®^ nutrition calculation program (Centro de Enseñanza Superior de Nutrición y Dietética. Universidad de Barcelona [21]. The reference values for minerals and vitamins dietary reference intake (DRI) in different ages are the updated tables of the National Academy of Sciences [22].

### 2.3. Nutrition Study

Sex, age, weight and height from every school child were recorded. Weight and height assessment were done in underclothes and shoes off. Weight was measured using an Año-Sayol^®^ scale (read range 0–120 kg and precision 100 g) and height was measured using a wall mounted rigid stadiometer (ranking 60–210 cm and with 0.1 cm precision). BMI Z-scores were calculated using the SEINAPTRACKER program (Medicalsoft Intercath, S.L. Universidad de Barcelona, 2007–2008). Reference growth curves and charts were the Centro Andrea Prader (Zaragoza, 2004) charts.

Results are shown as means (M) and percentages (%) with corresponding standard deviations (SD) of confidence intervals (95% CI). The SPSS version 20.0 (Chicago, Illinois, USA) program was used for statistics and analysis (Student’s T test, comparisons of proportions). Statistical significance was assumed for a p value lower than 0.05.

Note: As there were no statistically significant differences in results among sexes, they are shown together (consumption frequencies, nutrients and calorie intake and percentage contribution of food groups).

## 3. Results

### 3.1. Characteristics of the Sample

The average age of the surveyed students was 10.5 years (CI 95%: 10.3–11.7), and there were no statistically significant differences between both sexes. In the same way, there were no significant differences in mean values for weight (males: 38.8 ± 0.8 and females: 39.0 ± 0.7), height (males: 143 ± 0.6 and females: 142.8 ± 0.7) and BMI (males: 18.6 ± 0.1 and females: 18.42 ± 0.3). All survey respondents relayed to have lunch and dinner; however, 1.1% (*n* = 4), 5.9% (*n* = 21) and 3.1% (*n* = 11) referred not having breakfast, lunch or an afternoon snack, respectively.

### 3.2. Consumption Frequencies

As for breakfast, dairy products (91.5%), sweets, bakery and pastry (50%) and/or cereals (43%) and, to a lesser extent; fruits and natural juices (14%) and fats and oils (12%) were the food groups with a higher consumption. In addition, 72% and 15% of the respondents referred the addition of cocoa powder and sugar to milk, respectively. A glass of milk was the only intake for breakfast in 14% of the respondents.

As for mid-morning snack, bread (61%), together with cured meat (46%), fruits (12%) and yoghurt (11%) were the most consumed foods.

As for lunch, meat (74%), as well as cereals (67%), dairy products (45%), fruits (37%), legumes (30%) and vegetables (26%), tubers (18%) and fish (15%) were the most consumed foods. Fifty-two (52%) percent of surveyed students referred to the consumption of bread for lunch and 13% fried potatoes as garnish for meat or fish.

As for mid-afternoon snack, bread (78.2%), along with cured meat (42%), chocolate and/or cocoa butter (26%) and, to a lesser extent, yoghurt (13%) and fruits (8%) were the foods with a higher consumption.

As for dinner, dairy products (77%), meats (69%), cereals (60%), vegetables (24%), tubers (20%), eggs (19%), fruits (19%) and fish (15%) were the foods with a higher consumption. In addition, 53% of the surveyed students said to consume bread for dinner.

### 3.3. Nutrients and Calorie Intake

Average value of daily calorie intake was 2066.9 Kcal (CI 95%: 2040.2–2093.6). Table 1 sets out the average value for calorie intake and proportional calorie intake of every daily intake of the totality of the surveyed members of the sample, showing no statistical differences among both sexes. The highest calorie contribution corresponds to lunch (34.5%), followed by dinner (23.5%), breakfast (16%), mid-afternoon snack (14.5%) and, finally, mid morning snack (11.9%).

**Figure 1:**
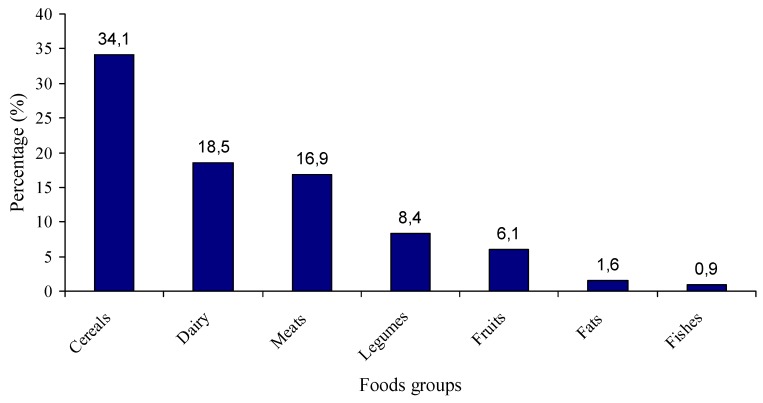
Percentage contribution of the different food groups to daily calorie intake.

Figure 1 displays the percentage contribution of the different food groups to daily calorie intake. Cereals (34%) dairy products (19%) and meats (17%) were responsible for approximately 70% of total calorie intake.

**Table 1:** Calorie intake (kilocalories) and calorie percentage contribution (%) of daily meals in the surveyed students.

Meal	Calorie intake (Kcal)	Calorie contribution (%)
	M (CI 95%)	M (CI 95%)
Breakfast	323.5 (313.9–333.1)	16.0 (15.5–16.5)
Mid-morning snack	253.8 (242.2–265.4	11.9 (11.4–12.4)
Lunch	734.2 (714.6–753.8)	34.5 (33.7–35.4)
Afternoon snack	298.1 (287.7–308.5)	14.5 (14.0–15.0)
Dinner	498.2 (480.3–516.1)	23.5 (22.8–24.2)

Table 2 exposes percentage contributions of the macronutrients and fatty acids to the total calorie intake in every respondent, so making a comparison with a healthy diet prototype. An excessive protein intake is observed mainly from animal origin, a deficient intake of carbohydrates and a higher than recommended intake of fatty acids. The daily average consumption of cholesterol was 315.6 ± 95.9 mg and total fiber 26.5 ± 13.5 g.

**Table 2:** Percentage contribution of immediate principles and fatty acids to daily calorie intake in the whole group.

Nutrients (recommended percentage)	Percentage (%)
Proteins (10%–15%)	20.3
Animal origin	64.5
Carbohydrates (50%–55%)	48.8
Total fat (30%–35%)	30.9
SFA (7%–10%)	12.6
MUFA (15%–20%)	10.9
PUFA (6%–10%)	3.74

Table 3 shows the average values of the intake of minerals and vitamins. The average value of calcium, iodine and Vitamins A, D and E was lower than recommended dietary allowances (RDA) for minerals and vitamins, respectively.

**Table 3:** Mineral and vitamin daily intake.

Nutrients	Total group M (SD)	DRI
Calcium (mg)	911.7 (174.1)	1300
Iron (mg)	18.3 (6.0)	8
Iodine (μg)	79.2 (22.6)	120
Magnesium (mg)	311.8 (83.3)	240
Zinc (mg)	10.5 (2.7)	8
Selenium (μg)	125.8 (39.6)	40
Phosphorus (mg)	1606.8 (294.0)	1250
Thiamine (mg)	1.7 (0.5)	0.9
Riboflavin (mg)	1.9 (0.5)	0.9
Niacin (mg)	39.0 (7.6)	12
Vitamin B_6_ (mg)	2.0 (0.6)	1.0
Folate (μg)	334.6 (150.2)	300
Vitamin B_12_ (mg)	5.8 (2.5)	1.8
Vitamin C (mg)	54.3 (34.3)	45
Vitamin A (μg)	463.6 (204.6)	600
Vitamin D (μg)	88.0 (66.3)	200
Vitamin E (mg)	3.8 (1.7)	11

### 3.4. Percentage Contribution of Food Groups

Table 4 shows the percentage contribution of the different food groups in daily macronutrients intake. The ingestion of proteins came mainly from meats (38%), cereals (20%) and dairy products (19.7%). Carbohydrates from cereals (55%), lipids from dairy products (36.9%) and meats (27.7%). SFA came mainly from dairy products (47.5%), meats (22.4%) and sweets, bakery and pastry (18.9%); cholesterol from meats (42.9%), eggs (21.4%) and dairy products (20.2%). Finally, fibre from legumes (37.8%), cereals (36.5%) and fruits (16.9%).

**Table 4:** Percentage contribution (%) of the different food groups to daily macronutrients intake.

Food group	Dairy	Cereals	Meats	Eggs	Vegetab	Legumes	Fruits	Fishes	Sweets	Fat
Proteins	19.7	20.8	38.0	2.0	0.5	11.5	0.7	2.9	3.9	-
CH	9.0	55.0	2.4	-	0.7	9.1	10.6	-	13.2	-
Fat	36.9	6.4	27.7	3.6	1.9	-	1.4	0.8	15.1	6.2
SFA	47.5	3.2	22.4	2.2	-	-	-	-	18.9	4.3
MUFA	32.4	3.3	33.5	4.8	-	-	1.8	-	14.5	7.8
PUFA	23.8	12.3	27.5	3.9	7.1	-	3.0	1.5	9.9	11.0
Cholest	20.2	-	42.9	21.4	-	-	-	5.4	8.5	1.6
Fibre	-	36.5	-	-	2.4	37.8	16.9	-	6.4	-

Vegetab: Vegetables; CH: Carbohydrates; Cholest: Cholesterol.

Table 5 shows the percentage contribution of the different food groups to the daily minerals’ intake. Calcium intake came mainly from dairy products (71.8%); iron from cereals (30.2%), meats (25.1%) and legumes (23.1%); iodine, from dairy products (43.7%); magnesium, from cereals (26.8%), legumes (25.5%) and dairy products (20.9%); zinc, from meats (43.5%); selenium, from cereals (62.4%). Finally, phosphorus from dairy products (35%) and meats (25.5%).

**Table 5:** Percentage contribution (%) of food groups to daily mineral intake.

Food group	Dairy	Cereals	Meats	Eggs	Vegetab	Legumes	Fruits	Fishes	Sweets	Fat
Ca	71.8	11.6	2.4	1.0	4.9	-	1.4	-	5.9	-
Fe	2.7	30.2	25.1	1.9	1.5	23.1	6.6	1.5	7.2	-
I	43.7	16.6	15.5	2.6	2.2	-	2.6	8.6	6.8	1.4
Mg	20.9	26.8	14.0	-	1.6	25.5	4.7	2.4	3.3	-
Zn	19.8	15.0	43.5	2.76	14.2	-	2.1	-	1.8	-
Se	5.1	62.4	14.2	1.2	4.3	-	2.1	7.1	3.6	-
P	35.0	15.2	25.5	2.2	14.0	-	1.6	2.2	4.4	-

Table 6 details the percentage contribution from the different food groups in daily vitamin intake. Thiamine (Vitamin B1) intake came mainly from meats (29.6%) cereals (27%) and legumes (17%); riboflavin (Vitamin B2) from dairy products (41.3%), meats (19.5%) and cereals (19.4%); niacin, from meats (42.5%) and cereals (25.6%); Vitamin B6, from meats (38%), cereals (21.3%) and legumes (17.6%); folate, from legumes (34.9%) and cereals (30.4%); Vitamin B12, from dairy products (38.3%) and meats (38.3%); Vitamin A, from dairy products (47.6%); Vitamin C, from fruits (54%) and cereals (18.4%); Vitamin D, from cereals (43.1%) and, finally, Vitamin E, from legumes (29.8%) and fruits (20.8%).

**Table 6:** Percentage contribution (%) of food groups to daily vitamin intake.

Food group	Dairy	Cereals	Meats	Eggs	Vegetab	Legumes	Fruits	Fishes	Sweets	Fat
B1	11.2	27.0	29.6	1.3	1.3	17.1	5.3	1.3	6.6	-
B2	41.3	19.4	19.9	3.5	7.0	-	3.5	1.0	5.0	-
Niacin	10.3	25.6	42.5	1.4	9.8	-	0.7	5.7	3.9	-
B6	9.9	21.3	38.0	1.0	1.9	17.6	5.9	1.9	2.8	-
Folate	9.4	30.4	5.3	3.4	4.3	34.9	4.8	1.2	6.3	-
B12	38.3	4.1	38.3	9.4	-	-	-	77	2.3	-
C	9.4	18.4	4.7	-	13.5	-	54.0	-	-	-
A	47.6	11.9	-	9.7	14.1	-	2.4	-	7.2	7.0
D	11.0	43.1	-	17.2	-	-	-	-	8.8	19.9
E	11.4	3.2	6.5	8.4	4.3	24.6	20.8	2.5	6.5	11.2

## 4. Discussion

Nutrition surveys based on recall are an optimal and widely used method in cross-sectional studies with descriptive purposes, as in this case [23]. A compendium of food pictures showing servings and sizes was used in order to facilitate the respondents to specify the quantity and/or size of the corresponding servings they had consumed in the previous three days. These pictures allowed both the surveyed students to identify the serving/size and the interviewer to estimate the consumed amounts [20]. These kinds of surveys have important methodological limitations we have tried to correct. On one hand, the option to include a 24-hour recall would have not considered adequately the intra individual variability in the sample; on the other hand, these surveys are somehow dependent on memory and, in addition, especially in this case, on the children’s ability to describe the consumed food; that is the reason why the surveyed children were students of 3rd–6th grade in primary education (9–12 years old).

At present day, the clinical validity of BMI as anthropometric parameter to define nutrition status in childhood and adolescence is admitted [24,25,26]. Owing to the variability in body composition throughout the pediatric age, specific reference charts are used for different ages and sex, being local reference charts—whenever available—recommended. This nutrition study has used as a reference the charts and tables from Ferrandez *et al.* (Centro Andrea Prader, Zaragoza 2004), which are widely spread and have proven utility in our environment.

Eating habits of surveyed scholars, all of them in normal nutritional status, reflected a dietary model that, even though it covered calorie needs for the corresponding age, it differed slightly from the Mediterranean prototype. On the whole, school kids consumed meats and derivatives almost every day and on a recurring basis. Meanwhile, the consumption of food of vegetable origin, such as vegetables, legumes and fruits, was considerably lower, and fish consumption was marginal. Dairy products and cereals consumption was relatively limited. However, sweets, bakery and pastry consumption was slightly high, being responsible for 13.2% of the whole of carbohydrates. However, the distribution of calorie intake along the five daily meals was adjusted to recommended proportional distribution.

An immediate consequence of the acquisition of this dietary pattern by schoolchildren in our environment is the evidence of a clear imbalance in percentage contribution of immediate principles to daily calorie intake. Fat intake, even though quantitatively sufficient since it represented 30.9% of total calorie intake, showed an excess in SFA to the detriment of MUFA and PUFA; in addition, cholesterol dietary intake, especially in males, exceeded the recommended 300 mg per day. Carbohydrate intake barely represented 48.8% of total calorie intake; this means, it did not get to the percentage contribution it should represent as a main energetic immediate principle at that age. However, protein intake slightly overtook the established recommendations, since it represented 20.3% of total calorie intake; furthermore, there was a clear imbalance between animal and vegetable origin, to the point that animal protein intake was responsible for two thirds of total protein intake. Another consequence of this dietary model is a deficient coverage in some minerals and vitamins; in fact, calcium, iodine, Vitamin A, D and E intake was below the established recommendations in both sexes. This means the characteristics of this dietary model do not match the basic concept of a balanced diet, since, despite an adequate energy intake, it does not guarantee sufficient nutrient intake in adequate quantity or proportion to get an optimal nutrition status.

Another characteristic of a balanced diet is the variety and diversity of foods it is made of. However, the dietary model of the surveyed students lacked this distinctive feature; in fact, the majority of nutrients were provided, with some exceptions, by the triad: dairy products, cereals and meats. When analyzing the percentage contribution of the different food groups in nutrient intake, we observe how the intake of proteins, SFA and cholesterol depended, in large measure, on meats and/or cured meat. Although meat is an essential element in any balanced diet as a source of high biological value proteins, minerals (iron, zinc and phosphorus), B-complex vitamins (thiamine, riboflavin, niacin, pyridoxine and cyanocobalamin), it is also a source of SFA (palmitic, stearic, myristic) and cholesterol. That is the reason why it is advisable to relegate lean meats and/or poultry consumption to 3–4 servings per week, and fatty meats and/or cured meats to occasional situations. Likewise, dairy products also contribute substantially to protein, saturated fat and cholesterol intake, besides representing the main source of calcium, iodine and Vitamin A. However, even though dairy products have a high content in SFA and cholesterol, their consumption should be increased mainly as yoghurts and cheese, with the aim to make up for the deficient calcium and, to a lesser extent, Vitamin A and iodine intake noticed in school kids; when having a normal nutritional status—as it happened in this case—low fat and/or modified fat composition dairy products could be consumed. Finally, it is remarkable how cereals contribute specially to calorie intake by means of high content in complex carbohydrates; in addition, they are an important source of proteins, which need to be complemented with other proteins of vegetable and/or animal origin, since they are low biological value proteins, as well as dietary fibre, minerals (iron, magnesium and selenium) and B-complex vitamins (thiamine, riboflavin, niacin, pyridoxine and folate). Therefore, at the same time meat consumption is moderated, cereal intake (breakfast, bread, rice, pasta) should be encouraged, in order to increase, on one side, its calorie percentage contribution and, on the other side, to average the origin of protein content in the diet. In addition, it would contribute to increasing calcium intake and compensating the hypothetical lower contribution of B-complex vitamins from meats.

Consumption of the rest of the food groups among surveyed students, except for sweets, bakery and pastry, was obviously lower and would explain, in large measure, the deficient intake in iodine and fat-soluble vitamins (A, D and E). For example, although nutritional value of vegetables as a source of macronutrients is limited, with the exception of tubers—which are rich in starch—they are rich in dietary fibre and Vitamins A, E and C. In this way, their daily intake would help correct the deficiency in vitamins of the dietary model in schoolchildren. Legumes should be given an appropriate consideration due to the high content in proteins, dietary fibre, iron and calcium, Vitamin E, as well as compensate the hypothetical lower intake of iron and B-complex vitamins obtained from meat. Fruits are a low calorie food group, but they stand out because of their content in dietary fibre and Vitamins A, C and E. That is why the daily or recurrent intake would contribute to a high intake in deficient Vitamins A and E, besides providing phytonutrients with antioxidant properties.

With regards to foods of animal origin and low frequency consumption as fish, it is to be noted that, in general, they have low calorie content but contain high biological value proteins—even higher than those from meat—and PUFA and provide water-soluble vitamins (thiamine, riboflavin and niacin), as well as fat-soluble vitamins (Vitamins A and D). In addition, sea fish and shellfish are the main natural source of iodine in our diet, being also a valuable source of calcium, phosphorus and iron. Therefore, its consumption should be promoted as an essential food in schoolchildren feeding. By doing so, a higher intake of iodine—together with iodized salt—PUFA and Vitamins A and D would be guaranteed and, consequently, it would improve the intake of these nutrients in schoolchildren.

The consumption of eggs among surveyed schoolchildren corresponds to the recommended frequency (no more than one per day and three per week). The fat content in the yolk is mainly SFA and MUFA and cholesterol and, in addition, the yolk is rich in minerals (iron, calcium, zinc and selenium) and water-soluble (thiamine, riboflavin and Vitamin B12) and fat soluble vitamins (Vitamins A and D). However, despite this moderated intake, they contribute noticeably to the cholesterol intake in schoolchildren diet.

Bakery and pastry intake was truly noticeable, especially in breakfast or afternoon snacks of schoolchildren. The nutritional value of pastry products is quite heterogeneous, due to the great variety of ingredients and proportions used. In general, energy value is very high owing to its composition of refined sugars and fat. For instance, the quantity of SFA depends on the type of vegetable oil used (palm oil or olive oil) and the amount of cholesterol depends on the origin of the fat (lard and butter) or the addition of some ingredients (eggs). What is recommended is its occasional ingestion and, therefore, it is advisable to substitute sweet, bakery and pastry with cereals and fruits, so increasing the intake of fibre, minerals and vitamins, as well as nutrients with functional properties, and reducing noticeably the intake of cholesterol and SFA.

This dietary pattern, together with the data provided by different authors, highlight a tendency in Spanish society—schoolchildren eat the available food in their houses—to join up to the new occidental dietary models, which show high intake of proteins and animal fat at the expense of complex carbohydrates [27,28,29,30,31,32,33,34,35,36].

As a strength of the study, we should mention the fact that a normal nutritional status among schoolchildren in our environment does not guarantee that their dietary model be healthy, since we find nutrition imbalance. However, the most relevant finding is that these nutrition deficiencies would remain corrected by increasing dairy products (milk, yoghurt, *etc.*), cereals (bread, rice, pasta, *etc.*), legumes, fruits and vegetables and fish (tuna, sardine, salmon, *etc.*) intake and reducing the intake of meat as well as promoting the use of olive oil as the exclusive culinary oil instead of other vegetable oils. In this way, we could guarantee a sufficient intake of all vitamins and minerals that are deficient in the dietary model of the surveyed schoolchildren.

An important conclusion emerged from this work: it is imperative to design nutrition education programs with the goal of letting the general population and schoolchildren in particular become aware and prepare for healthy dietary habits. In order to do this, public authorities should coordinate human and/or material resources enough to attempt to maintain our traditional feeding habits and make them compatible with the new lifestyle in modern societies, encouraging nutritional counselling in primary health care and developing nutrition education programs in educational institutions. This way, schoolchildren would have excellent means to prevent disease and promote health after ending compulsory education.
